# Nifedipine Ameliorates Cellular Differentiation Defects of Smn-Deficient Motor Neurons and Enhances Neuromuscular Transmission in SMA Mice

**DOI:** 10.3390/ijms24087648

**Published:** 2023-04-21

**Authors:** Rocio Tejero, Mohammad Alsakkal, Luisa Hennlein, Ana M. Lopez-Cabello, Sibylle Jablonka, Lucia Tabares

**Affiliations:** 1 Department of Medical Physiology and Biophysics, School of Medicine, University of Seville, 41009 Seville, Spain; rtejeronavarro@gmail.com (R.T.);; 2 Institute of Clinical Neurobiology, University Hospital Würzburg, 97078 Würzburg, Germany

**Keywords:** spinal muscular atrophy, motor neurons, synaptic transmission, neuromuscular junction, calcium channels, nifedipine, growth cone, axons, synaptic vesicles, postsynaptic potentials

## Abstract

In spinal muscular atrophy (SMA), mutations in or loss of the *Survival Motor Neuron 1* (*SMN1*) gene reduce full-length SMN protein levels, which leads to the degeneration of a percentage of motor neurons. In mouse models of SMA, the development and maintenance of spinal motor neurons and the neuromuscular junction (NMJ) function are altered. Since nifedipine is known to be neuroprotective and increases neurotransmission in nerve terminals, we investigated its effects on cultured spinal cord motor neurons and motor nerve terminals of control and SMA mice. We found that application of nifedipine increased the frequency of spontaneous Ca^2+^ transients, growth cone size, cluster-like formations of Cav2.2 channels, and it normalized axon extension in SMA neurons in culture. At the NMJ, nifedipine significantly increased evoked and spontaneous release at low-frequency stimulation in both genotypes. High-strength stimulation revealed that nifedipine increased the size of the readily releasable pool (RRP) of vesicles in control but not SMA mice. These findings provide experimental evidence about the ability of nifedipine to prevent the appearance of developmental defects in SMA embryonic motor neurons in culture and reveal to which extent nifedipine could still increase neurotransmission at the NMJ in SMA mice under different functional demands.

## 1. Introduction

SMA is the most frequent neurodegenerative disease affecting motor neurons in children. Its incidence is 1 in 6000–10,000 newborns, with a carrier frequency of 1:40–50. It is an autosomal-recessive inherited disease caused by biallelic mutations in the survival motor neuron 1 (*SMN1*) gene [1]. The gene product, the SMN protein, is involved in snRNP assembly and splicing, mRNA transport, local translation, cytoskeleton dynamics, and others [2,3]. Although SMA is ubiquitously expressed, it is particularly abundant in lower spinal cord motor neurons. Clinically, SMA is characterized by loss of alpha motor neurons and predominantly axial and proximal muscle weakness [4]. Four clinical phenotypes have been described based on the age of onset of symptoms and the degree of motor dysfunction [4,5,6,7]. Type I is the most severe phenotype, affecting about 50% of SMA patients. Until recently, type I infants could not sit or walk and would die before reaching two years of age. However, in the last years, SMN-increasing therapies have been used (gene therapy, antisense oligonucleotides, and small molecules) with good results in most patients, if applied presymptomatically [8,9,10,11]. However, if the treatment is delayed, motor milestones could be retarded or not reached. Therefore, developing SMN-independent therapies could contribute to alleviating the disease’s pathophysiology.

In SMA mouse models, embryonic motor neurons in culture show obvious cellular differentiation defects, such as altered axon lengths and smaller growth cones than control motor neurons. They also display reduced β-actin levels and F-actin assembly [12,13,14] that, in turn, diminishes Cav2.2 accumulations in growth cones but not in cell bodies [13,14]. Decreased Cav2.2 cluster-like formations correlate with spike-like Ca^2+^ transient frequency reduction at distal axons and growth cones [13,15] that correspond to altered axon elongation on laminin-221/211, the motor endplate-specific isoforms. In ex vivo experiments in mouse SMA models, functional studies have shown that motor nerve-evoked neurotransmission is significantly decreased, as evidenced by the small size of their evoked endplate potential (EPPs) and reduced quantal content (*m*) compared to controls [16,17,18,19]. The number of docked vesicles is also reduced, as assessed by electron microscopy and electrophysiological analysis of the readily releasable pool (RRP) of vesicles [18]. In addition, SMA motor nerve terminals display a reduction in Cav2.1 channel clusters compared to controls [19,20].

Two P/Q-type (Cav2.1) and N-type (Cav2.2) Ca^2+^ channel gating modifiers have recently been tested in SMA models with interesting results. For example, R-Roscovitine, which is also a cyclin-dependent kinase inhibitor [21,22], improved neurotransmission, terminal size, and Cav2.1 channel density in SMA motor nerve terminals. R-Roscovitine also improves Ca^2+^ signaling and development in SMN-deficient motor neurons in culture [20]. GV-58, an R-Roscovitine derivate that also increases the opening time of Cav2.1 and Cav2.2 channels, significantly enhances neuromuscular transmission and muscle strength in SMA mice [23]. 

Besides P/Q- and N-type voltage-gated Ca^2+^ channels (VGCCs), L-type Ca^2+^ channels (Cav1.2) are expressed in motor nerve terminals and modulate evoked neurotransmission in immature NMJs [24,25]. L-type Ca^2+^ channels are also expressed in neurons, facilitating Ca^2+^ entry to the cytosol during electrical activity. At axonal growth cones, L-type channels, together with other Ca^2+^ and K^+^ channels, modulate the size and signaling of the nerve endings [26]. In some pathological situations, however, dysregulation of cellular Ca^2+^ homeostasis occurs, and L-type Ca^2+^ channels may play a role. 

Experimental evidence shows that chronic exposure to nifedipine, a classical dihydropyridine (DHP) antagonist of L-type Ca^2+^ channels, reduces the death of mSOD1^G93A^ motor neurons [27] and protects against AMPA-induced mitochondrial stress [28]. Nifedipine also enhances functional motor unit expansion (sprouting) and muscle function in the absence of the presynaptic neural cell adhesion molecule (NCAM) [29]. Applying nifedipine to motor nerve terminals increases evoked neurotransmission in newborn rats and mice and adult regenerating terminals from frogs and mice [24,25]. Nifedipine also increases spontaneous release in motor nerve terminals by mobilizing intracellular Ca^2+^ stores [30]. However, despite all these potential beneficial effects of nifedipine on altered neurons, its effects on SMA motor neurons and nerve terminals have not been explored. 

Here, we studied the impact of nifedipine on the cellular differentiation of cultured embryonic spinal cord motor neurons from control and SMN-deficient mice. We also investigated the capability of nifedipine to modulate neurotransmission in SMA (SMNΔ7) mice ex vivo. We show that nifedipine is beneficial in alleviating both motor neuron development defects and NMJ function alterations in SMA models. This study could be the basis for further research to assess whether nifedipine is helpful as a complementary therapy to actual SMN restoration treatments.

## 2. Results

### 2.1. Nifedipine Treatment Leads to Proper Cellular Differentiation of SMN-Deficient Motor Neurons by Restoring Altered Cav2.2 Cluster Formation and Spontaneous Ca^2+^ Transients at Growth Cones

**Figure 1:**
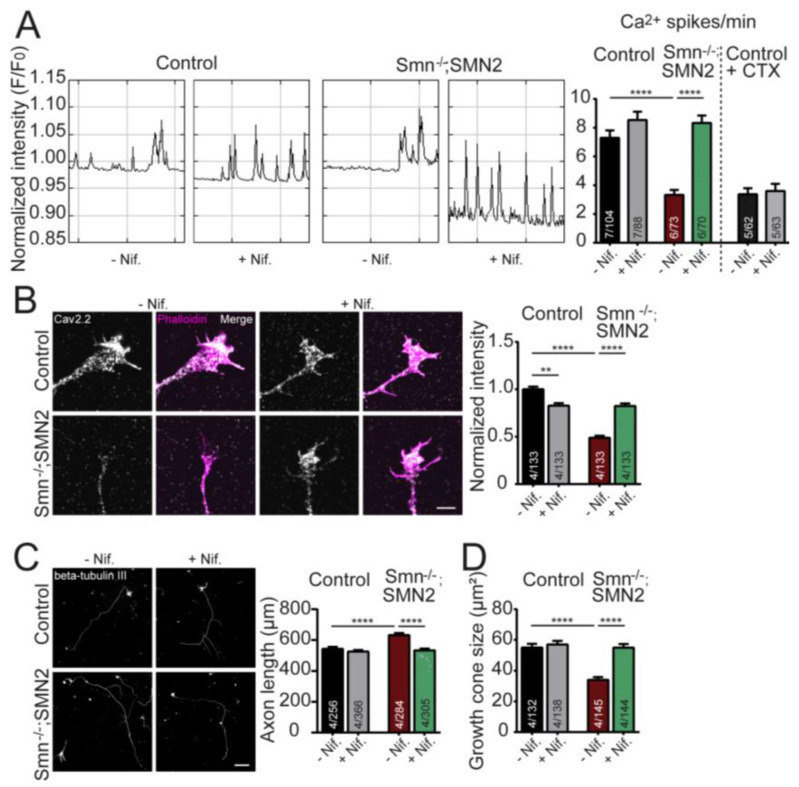
Nifedipine increases the frequency of Ca^2+^ transients and improves Cav2.2 cluster-like formation at the growth cones of embryonic motor neurons cultured on laminin-221/211. (**A**) Representative plots of Oregon Green™ 488 BAPTA-1 fluorescent changes over time, indicating spontaneous Ca^2+^ spikes of control and *SMN^−/−^;SMN2^+/+^* motor neurons untreated and treated with nifedipine (+Nif.). Quantification of spontaneous Ca^2+^ spikes per min in control and *SMN^−/−^; SMN2*^+/+^ growth cones untreated and Nif. treated, as well as control growth cones that were additionally treated with ω-Conotoxin MVIIA (CTX) (ANOVA Kruskal–Wallis, **** *p* ≤ 0.0001). (**B**) Growth cones from control and *SMN^−/−^;SMN2^+/+^* motor neurons untreated and Nif. treated, stained against Cav2.2 (gray) and F-actin (Phalloidin, magenta). Normalized mean gray values of Cav2.2 (ANOVA Kruskal–Wallis, ** *p* ≤ 0.01; ****; *p* ≤ 0.0001). Scale bar: 5 µM. (**C**) Motor neurons from control and *SMN^−/−^;SMN2^+/+^* mice untreated and treated with Nif., stained against β-tubulin III (gray). Scale bar: 100 µM. Nif. treatment in *SMN^−/−^;SMN2^+/+^* motor neurons rescued axon length (control: 542.7 ± 12.4 µm; control + Nif.: 524.6 ± 9.9 µm; *SMN^−/−^;SMN2^+/+^*: 631.7 ± 13.5 µm; *SMN^−/−^;SMN2^+/+^* + Nif.: 532.3 ± 12.1 µm; ANOVA Kruskal–Wallis test, **** *p* ≤ 0.0001). (**D**) Nif. treatment increases growth cone size in SMN-deficient motor neurons (control: 54.97 ± 2.42 µm^2^; control + Nif.: 56.99 ± 2.37 µm^2^; *SMN^−/−^;SMN2^+/+^*: 34.07 ± 1.83 µm^2^; *SMN^−/−^;SMN2^+/+^* + Nif.: 54.95 ± 2.34 µm^2^; ANOVA Kruskal–Wallis test, **** *p* ≤ 0.0001). Data are presented as bar plots. Bars represent the mean ± SEM. Groups are labeled as *N/n* where *N* is the number of independent experiments and *n* is the number of individual growth cones analyzed.

The effect of nifedipine on the frequency of spontaneous Ca^2+^ transients in cultured embryonic motor neurons was analyzed within the axon terminals using the Oregon Green 488 BAPTA-1 Ca^2+^ indicator. As previously described, SMN-deficient motor neurons displayed a significantly decreased frequency of spontaneous Ca^2+^ transients at growth cones compared to control neurons. Nifedipine treatment resulted in a significant increase in the frequency of the Ca^2+^ transients in SMN-deficient growth cones. At the same time, the effect on control neurons was only mild and did not reach statistical significance (Figure 1A). To verify whether N-type Cav2.2 channels mediated the Ca^2+^ influx, motor neurons were treated with ω-Conotoxin MVIIA (CTX), together with nifedipine, over the entire culture period. Treating control motor neurons with CTX, a specific blocker of N-type VGCCs, resulted in a significant reduction of Ca^2+^ transients in growth cones to a level comparable to SMN-deficient motor neurons (Figure 1A). 

We further checked whether nifedipine treatment ameliorated the Ca^2+^ channel clustering defects observed in SMN-deficient motor neurons. Therefore, the distribution of the N-type Cav2.2 channels was investigated within the axon terminals of cultured SMA motor neurons treated with 10 µM nifedipine for the entire culture period (five days in vitro, DIV 5). The immunocytochemical analysis confirmed that signal intensities of Cav2.2 channels were significantly reduced in growth cones of SMN-deficient motor neurons compared to controls (Figure 1B). However, nifedipine treatment resulted in a marked improvement in localization of Cav2.2 channels in SMN-deficient motor axon terminals (Figure 1B,C). In contrast, control motor neurons showed slightly but significantly reduced N-type VGCCs upon nifedipine treatment during the entire culture period (Figure 1B). 

Proper formation of Cav2.2 clusters is essential for spontaneous Ca^2+^ transients during neuronal development [13]. Based on the observation that nifedipine treatment significantly improves Cav2.2 cluster-like formations and the frequency of spontaneous Ca^2+^ transients in motor axon terminals of SMA motor neurons, we hypothesized that other cellular differentiation defects are beneficially affected as well. Thus, the axonal outgrowth of nifedipine-treated motor neurons was examined at DIV7. As previously shown, the axon length of SMA motor neurons was significantly different from those observed in control motor neurons [13]. However, nifedipine treatment significantly decreased the axon elongation of SMA motor neurons on laminin-221/211 control levels (Figure 1C). Similarly, the growth cone size was markedly increased upon nifedipine treatment of SMA motor neurons at DIV5 (Figure 1D). In contrast, in control motor neurons, the addition of nifedipine did not affect growth cone size and axon elongation, respectively (Figure 1C,D). 

### 2.2. Nifedipine Increases Neurotransmission in Motor Nerve Terminals of Control and SMA Mice

Nifedipine increases neurotransmission in rat, mouse, and frog motor nerve terminals in a Ca^2+^-dependent manner during postnatal maturation [24,25]. Here, we investigated whether nifedipine could do so at mouse motor nerve terminals of the SMA, highly vulnerable muscle transversus abdominis (TVA) at a time (postnatal day 9–11, P9–11) in which the electrophysiological alterations were already significant [18,31]. 

**Figure 2:**
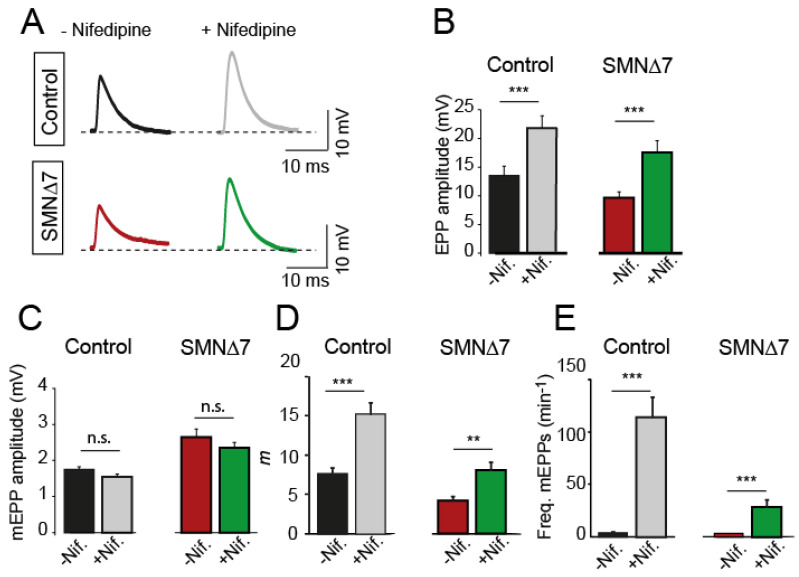
Secretion increases in the presence of nifedipine in motor nerve terminals from postnatal control and SMA mice. (**A**) Representative EPP recordings in the absence and the presence of nifedipine in control (top) and SMA (bottom) mice. (**B**–**E**) Mean EPP amplitudes (**B**), mEPP amplitudes (**C**), quantal content (*m*) (**D**), and mEPP frequencies (**E**) in the absence and presence of nifedipine in both genotypes. The numbers in the bars indicate the recorded fibers. N = 5 mice per genotype. Mann–Whitney U and two-tailed Student’s t (quantal content) -tests; *** *p* < 0.0005; ** *p* < 0.005.

Ex vivo application of 50 µM nifedipine to the bath solution increased mean EPP amplitudes in both genotypes similarly: ~1.9-fold in control mice (33 and 38 terminals, 7 mice, P_Mann-Whitney_ = 0.003) and ~1.8-fold in SMA mice (24 and 28 terminals, 4 mice; P_Mann-Whitney_ = 0.0003) (Figure 2A,B). In contrast, nifedipine did not affect the mean amplitudes of miniature EPPs (mEPPs) (Figure 2C), indicating that the potentiating effect on the EPP amplitude was presynaptic. As expected from the above results, the quantal content was about twice as high in the presence of nifedipine than in its absence, both in control (P_Mann-Whitney_ = 0.0002) and SMA (P_Mann-Whitney_ = 0.012) terminals (Figure 2D). Nifedipine also greatly enhanced the frequency of spontaneous neurotransmission in control (~28-fold) and SMA (~8.6-fold) terminals (Figure 2E). The vehicle alone (0.1% DMSO) did not produce any change in the above parameters [20]. At a lower nifedipine concentration (10 µM), analyses of neurotransmission did not reveal changes in the neuromuscular release, except for a slight increase in the frequency of spontaneous events in control (~2.2-fold), but not in SMA mice.

### 2.3. The Effect of Nifedipine on Neurotransmission Is Independent of L-Type Ca^2+^ Channels

We investigated whether the potentiation effect of nifedipine in postnatal terminals could be mediated by L-type Ca^2+^ channel blockage, as proposed in adult mice’s newly formed motor nerve terminals after mechanical nerve compression [32,33].

**Figure 3:**
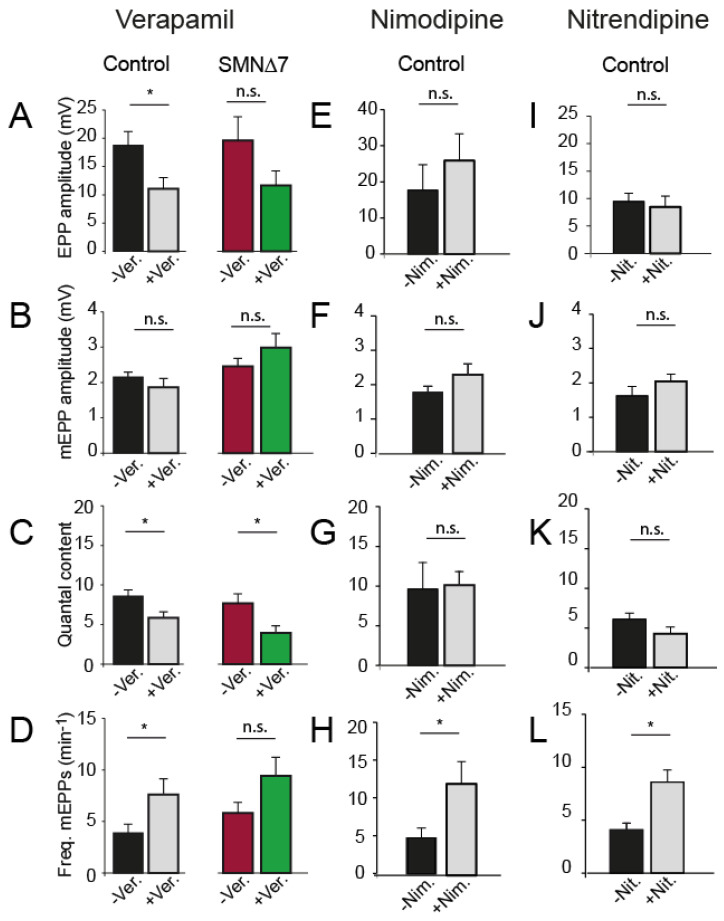
Nifedipine increases neurotransmission independently of its blocking effect on Ca^2+^ entry through L-type Ca^2+^ channels in postnatal mice. (**A**–**D**) Effect of verapamil (Ver., 3 µM), (**E**–**H**) nimodipine (Nim., 10 µM), and (**I**–**L**) nitrendipine (Nit., 50 µM) on EPP size (**A**,**E**,**I**), mEPP size (**B**,**F**,**J**), quantal content (*m*) (**C**,**G**,**K**), and mEPP frequency (**D**,**H**,**L**) in control terminals, except for verapamil that was also tested in SMA terminals (* *p* < 0.05; two-tailed Student’s *t*-test).

We first examined the effect of verapamil, a non-DHP L-type channel blocker, on secretion. Verapamil (3 µM) reduced the EPP size (Figure 3A) but not the mEPP amplitude in both genotypes (Figure 3B). Accordingly, verapamil decreased the quantal content (32% in control; *p* = 0.01, two-tailed Student’s *t*-test, and ~48% in SMA motor nerve terminals; *p* = 0.03; two-tailed Student’s *t*-test) (Figure 3C). The frequency of spontaneous release events slightly increased in control (~2-fold), but less in SMA mice (Figure 3D).

Next, we investigated whether two DHP L-type Ca^2+^ channel antagonists, nimodipine (10 and 50 µM) and nitrendipine (1, 25, and 50 µM), have a similar effect on secretion than nifedipine and found no change in evoked release at any concentration. Figure 3 shows the results obtained with 5 and 10 µM nimodipine (17 fibers, 3 mice) and 25–50 µM nitrendipine (20 fibers, 3 mice). Only spontaneous release was slightly (~2-fold) increased in the presence of either drug. Together, these results indicated that the blockage of L-type channels likely did not significantly mediate the nifedipine synaptic transmission potentiation in control and SMA mice. 

### 2.4. Nifedipine Increases the RRP Size in Controls but Not in SMA Terminals

**Figure 4:**
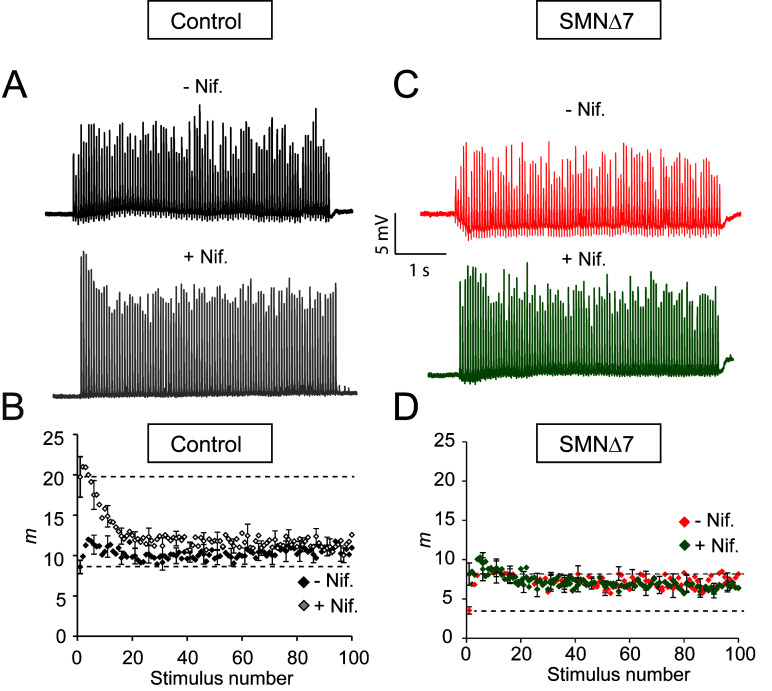
Nifedipine does not increase the size of the RPP in the SMA terminals. (**A**,**C**) Representative examples of EPP recordings during 20 Hz, 5 s trains in control (**A**) and SMA (**C**) terminals before and after the application of nifedipine. (**B**,**D**) Mean quantum content (*m*) in control (**B**) and SMA (**D**) mice in the absence and presence of nifedipine.

Since nifedipine potentiated secretion at low-frequency stimulation in both genotypes, we also examined whether this drug increases neurotransmission during high-frequency nerve stimulation (20 Hz trains of 5 s duration) in control and SMA motor nerve terminals. In controls, nifedipine increased EPP amplitudes, as illustrated in the representative examples shown in Figure 4A. Interestingly, nifedipine changed the typical postnatal exocytic response to an adult-like response, namely, initial large EPP amplitudes that progressively fell to a plateau of approximately one-third of the peak amplitude [34,35,36,37]. The plateau represents a constant balance between consumption and recruitment of vesicles during the train.

The plot of the average quantal content values versus stimulus number before and after nifedipine (27 and 28 fibers, respectively, 5 muscles) confirmed that the significant increment occurred during the first second of stimulation (Figure 4B), which corresponds to the time at which ~95% of the quanta come from the RRP [36,38]. On average, cumulated release after one second of stimulation in the absence of the drug was 213.1 ± 18 quanta versus 326.3 ± 28.9 quanta in the presence of nifedipine in control terminals (P_Mann-Whitney_ = 0.0022). Thus, nifedipine improved synaptic strength by increasing the size of an RRP. Interestingly, nifedipine did not alter synaptic vesicle replenishment, as indicated by the similar plateau amplitudes in the absence and presence of the drug. 

In contrast, nifedipine did not increase the RRP fullness in SMA terminals (Figure 4C,D; 21 fibers, 5 muscles). However, the first shock elicited a significantly larger response (Figure 4D, lower discontinuous line). Thus, the amplitude ratio between the second and the first EPP (pair-pulse facilitation) was reduced in the presence of nifedipine, indicating that the probability of release was already high during the first shock, in agreement with the high rate of spontaneous events during low-frequency stimulation. The lack of a net effect of nifedipine on the RRP is consistent with, among others, the reduction in docked vesicles [16], active zones [18], synaptotagmin 2 [19], and SNARE complexes [39] in SMA motor nerve terminals. Thus, the present data may reflect the upper functional limit during high electrical stimulation. 

### 2.5. The RRP Pool Size Is Insensitive to Nifedipine in Adult Motor Nerve Terminals

**Figure 5:**
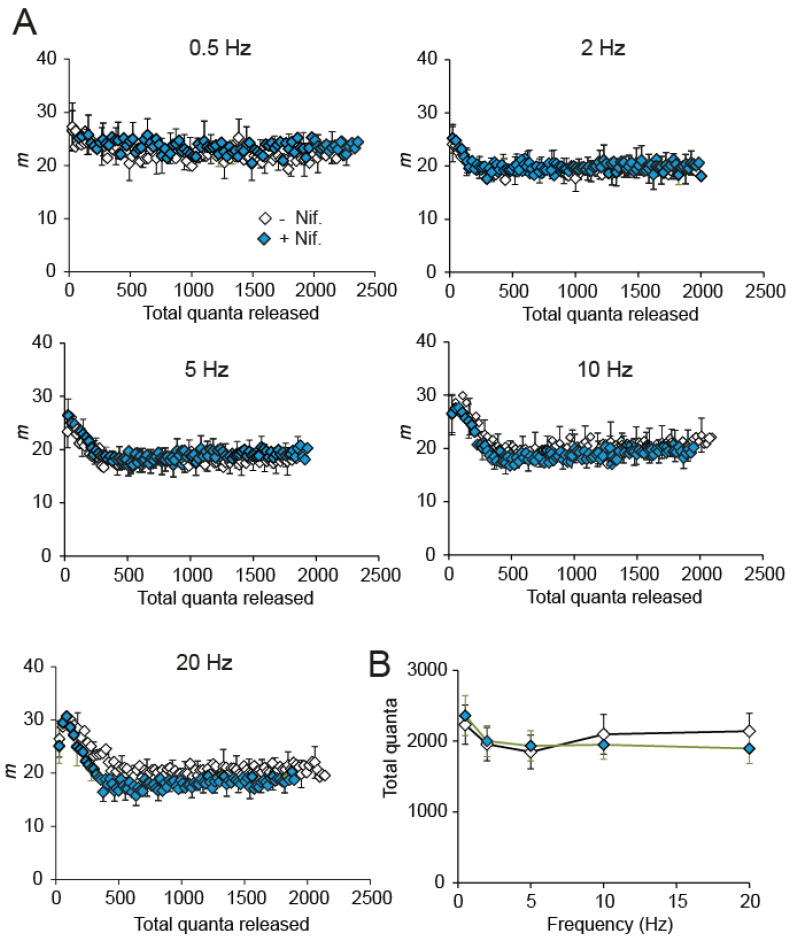
Evoked release in adult motor nerve terminals is insensitive to nifedipine. (**A**) Average quantal releases during trains of 0.5–20 Hz were indistinguishable without (black symbols) or with (green symbols) nifedipine. (**B**) Plot of the average quanta released during 100 shocks versus stimulation frequency in the absence and the presence of nifedipine.

Since the electrophysiological response to nifedipine during high-frequency stimulation in immature control terminals (Figure 4A,B) recalls the increased and reliable response in adult mice [36,40], we explored whether nifedipine was still able to enhance evoked and spontaneous secretion in adult mice. We found that the average quantal releases during trains of 0.5–20 Hz were indistinguishable with or without nifedipine (Figure 5A,B). In the same line, the nifedipine effect on spontaneous release frequency was relatively modest (~3.27-fold; P_Mann-Whitney_ = 0.0013). These results are consistent with previous findings of no effect of nifedipine in adult NMJs [24]. They suggest important changes in the modulation mechanism of the synapse with age. 

### 2.6. The Effect of Nifedipine on the RRP Is Calmodulin-Independent

Since nifedipine positively modulates synaptic strength by increasing the RRP (Figure 4), which indicates enhanced vesicle priming, we explored whether the Ca^2+^-CaM complex participates in this response. Modulation of priming is a Ca^2+^-dependent process that may affect the RRP effective size [40], vesicle refilling rate during repetitive stimulation, or both. Indeed, it has been shown that CaM plays a role in replenishing synaptic vesicles during high-frequency repetitive stimulation [41,42]. For example, the plateau amplitude in mice expressing a mutant form of the synaptic vesicle priming protein Munc13-1, which cannot bind CaM, is significantly lower than in wild-type mice [43]. 

**Figure 6:**
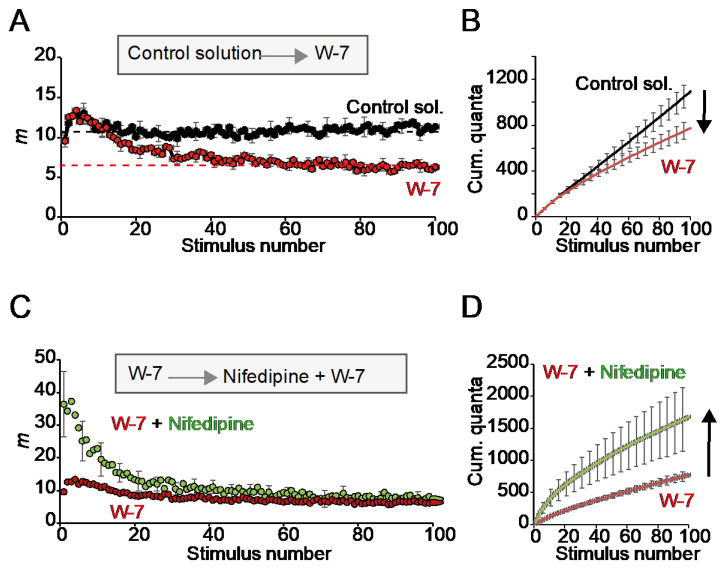
Effect of W-7 on secretion during repetitive high-frequency stimulation. (**A**) Average quantal content (*m*) in the absence (control solution) and presence of the CaM inhibitor W-7. Observe the increases in short-term depression (STD). (**B**) Mean cumulative release from recording in (**A**). (**C**) Average quantal content in the presence of W-7 and after adding nifedipine. (**D**) Mean cumulative release from recording in (**C**). The arrows represent the directions and amplitudes of the changes.

Then, we examined the effect of the CaM inhibitor W-7 in the absence and presence of nifedipine. Incubation of the preparation with W-7 (10 µM) alone did not significantly change release during the first second of stimulation. Still, it decreased the plateau amplitude by about half (Figure 6A). The resulting rise in short-term depression (STD) resulted in a ~29.3% reduction in cumulative release after 100 stimuli (Figure 6B; 18 fibers per condition). These results indicate that CaM inhibition did not affect the resting RRP size but attenuated vesicle replenishment. 

Next, we investigate whether nifedipine lost its potentiation effect on the RRP if the preparation was preincubated with W-7. Remarkably, nifedipine still increased the RRP in the presence of the CaM inhibitor but did not restore the plateau amplitude (Figure 6C,D). These results indicate that, in our preparation, nifedipine can operate in the absence of or under little activation of CaM and suggests different sensitivities in Ca^2+^-dependent priming at rest and during repetitive stimulation. 

In summary, nifedipine restored the reduced spontaneous calcium influx through N-type voltage-gated Ca^2+^ channels in embryonic SMN-deficient motoneurons by increasing cluster formation of the channels. These increased spontaneous Ca^2+^ transients correlated with enhanced cellular differentiation in terms of axonal growth. Moreover, our ex vivo studies showed that nifedipine improves low-frequency evoked and spontaneous neurotransmission in SMN-deficient motor nerve terminals.

## 3. Discussion

Compounds that decrease morphological and functional alterations in SMA models, such as 8-CPT-cAMP, R-Roscovitine, and GV-58 [13,20,44], may help to decipher aspects of the complex pathophysiology of SMA. The present study aimed to investigate the impact of nifedipine in the cellular differentiation of SMN-deficient motor neurons and neurotransmission at the NMJ in SMA mice. Our findings can be summarized as follows: (*i*) Chronic application of nifedipine alleviates morphological and functional defects in growth cones and axons from SMN-deficient motor neurons in vitro, (*ii*) nifedipine facilitates low-frequency evoked and spontaneous neurotransmission in motor nerve terminals of control and SMA mice during the second postnatal week, independent of its blocking effect on Ca^2+^ entry through L-type VGCCs, (*iii*) in adult motor terminals, nifedipine does not potentiate ACh release, (*iv*) nifedipine increases the RRP size in control but not in SMA mice at high stimulation frequency. The drug does not modify STD in either genotype, and (*v*) CaM effectively regulates STD but only slightly changes the RRP size. We now discuss the main findings of our study.

### 3.1. Nifedipine Beneficially Regulates the Cellular Differentiation of SMN-Deficient Motor Neurons

Alterations in the interaction between Ca^2+^ channels and the motor endplate-specific isoforms of laminin containing the β2-chain play an important role in the pathogenesis of SMA. The β2-chain interacts directly with the α1 subunit of Cav2.1 and Cav2.2 channels, leading to the clustering of these channels and initiation of presynaptic differentiation and maintenance of active zones [13,45]. Note that the β2-chain also interacts with Cav1.2, but to a lesser degree [45]. Gene knockout of the β2-chain of laminin or Cav2.1 channels leads to severe neuromuscular degeneration in mice [45,46]. SMN-deficient motor neurons cultured on the laminin endplate-specific isoforms (laminin-221/211) have decreased Cav2.2 channel accumulations at the growth cone, which correlates with a reduced frequency of local Ca^2+^ transients [13]. 

Our present results show that chronic exposure to nifedipine leads to increased frequency of spontaneous Ca^2+^ influx through Cav2.2 channels in growth cones in SMN-deficient motor neurons (Figure 1A), as we have already seen by the application of R-Roscovitine in SMA motor neurons [20]. The mechanisms by which both drugs produce the same effect are still unknown. Interestingly, they have opposite effects in VGCCs; while nifedipine blocks L-type Ca^2+^ channels [47], R-Roscovitine prolongs the opening time of N- and P/Q-type Ca^2+^ channels [22,48]. However, both drugs block a variety of K^+^ channels. For example, nifedipine inhibits Kv1.2 channels in motor neurons [49] and R-Roscovitine inhibits Kv1.3, Kv2.1, Kv4.4, and hERG (Kv11.1) channels [50,51]. Partial inhibition of outward K^+^ currents may lower the spontaneous Ca^2+^ transient generation threshold and increase global [Ca^2+^]_i_. Additionally, nifedipine may increase Ca^2+^ release from intracellular stores at nerve terminals, favoring the production of Ca^2+^ transients [52]. Further observations of our study showed that nifedipine application beneficially affects cellular differentiation on laminin-221/211 by reducing the axon length and increasing the growth cone sizes (Figure 1). This, in turn, corresponds to proper cluster-like formations of N-type VGCCs (Figure 1). Whether this effect is mediated by the increase in Ca^2+^ influx in primary cultured SMN-deficient motor neurons requires further work. 

### 3.2. Age-Dependent Nifedipine Potentiation of Evoked and Spontaneous Neurotransmission at the NMJ

The potentiating effect of nifedipine on evoked neurotransmission at the NMJ is age-dependent, being high in embryonic and newborn mice and absent in adults [24]. Interestingly, the presynaptic potentiation reappears after a lesion in regenerating adult mouse and frog NMJs [24,32,33]. The sensitivity to the drug is also age dependent. For example, 1 µM is effective at embryonic motor terminals [24] but not at P0–4 [25]; an amount of 10 µM nifedipine is effective at P0–4 but not at P5–11 [25]. In our experiments (P9–11), 50 µM nifedipine was efficient, but not 10 µM. These results suggest that the mechanisms responsible for the nifedipine effects at the NMJ disappear as the synapse matures. However, the precise molecular mechanisms participating in it are not entirely understood yet. 

Remarkably, besides increasing evoked release, nifedipine elevates spontaneous release in a dose-dependent manner. For example, in rat brain supraoptic neurons, nifedipine increases the frequency of miniature synaptic currents with a maximum response at 50 µM [53]. In neonatal rat phrenic-diaphragm nerve terminals, nifedipine augmented mEPP frequency up to 56-fold, while nitrendipine or isradipine, two Ca^2+^ channel blockers of the DHP class, had no effect [30]. Our results agree with these findings since spontaneous release frequency increased ~28-fold with nifedipine (Figure 2E) and much less, about two-fold, with other L-type blockers (Figure 3D). Most likely, nifedipine increases spontaneous release by mobilizing Ca^2+^ release from intracellular stores since the effect is independent of extracellular Ca^2+^, is abolished by depleting intracellular Ca^2+^ stores with thapsigargin, and is partially inhibited by ryanodine [30]. Interestingly, since other L-type blockers do not mimic this effect, it is suggested that nifedipine binds to a site other than the conventional DHP binding site. In accordance with it, our experiments showed that nifedipine increased both evoked and spontaneous release, but nimodipine, nitrendipine, and verapamil did not potentiate evoked release and only exerted a small effect on mEPP frequency. Release of Ca^2+^ from subsurface cisternae near exocytic sites may affect evoked release [52], particularly in immature synapses in which coupling between Ca^2+^ channels and release sites, and Ca^2+^ buffering capacity, might be low [54,55]. In addition, molecular developmental changes in protein isoforms and expression levels may also contribute to the different sensitivity of synapses to nifedipine with age.

### 3.3. The Multiple Targets of L-Type Ligand Drugs and Neurotransmission

Neurotransmission is modulated at central and peripheral synapses by different mechanisms. At the NMJ, autoregulation is mainly mediated by ACh activation of presynaptic nicotinic and muscarinic cholinergic receptors, which activate different intracellular pathways [56,57]. Activation of nicotine nerve ACh receptors (nNAChRs) predominantly produces neurotransmitter release autoinhibition at the NMJ with the possible participation of L-type VGCCs [24]. 

Although nifedipine is an L-type channel antagonist and increases neurotransmission in motor nerve terminals [24,25,32,33], the mechanism(s) responsible for this effect is not completely clear yet. Experimental evidence indicated that nifedipine also binds to several Kv channels [49,58] and nNAChRs [57]. Thus, nifedipine may directly block L-type channels but also reduce their activation through the blockade of nNAChRs, which may relieve the autoinhibitory mechanism of neurotransmitter release. In addition, nifedipine’s binding and blocking of Kv channels may prolong the nerve action potential, increasing Ca^2+^ influx through VGCCs and, subsequently, neurotransmitter release. Similarly, Ca^2+^ release from intracellular stores by nifedipine may enhance neurotransmission, mainly if Ca^2+^ comes from subplasmalemmal cisternae near exocytic sites [52]. Thus, it would be interesting to determine in upcoming studies which mechanisms prevail at NMJ. 

### 3.4. Is Nifedipine a Good Candidate for Complementary Therapy in Motor Neuron Diseases?

Although inducing proper motor neuron development and maturation and supporting neurotransmission are desirable goals and could be considered for additional therapy for SMA and other motor neuron diseases, modulating Ca^2+^ channels by small molecules may carry some side effects on the whole organism. Therefore, the possible risks of such molecules must always be closely monitored in preclinical studies and beyond.

## 4. Materials and Methods

The methods listed here aimed to measure the effect of nifedipine on motor neuron differentiation in vitro and on synaptic function ex vivo in control and SMA mice. The calcium imaging technique was used for measuring Ca^2+^ oscillation (spikes) frequency at the growth cone and the immunocytochemistry techniques were used to quantify the growth cone size, Cav2.2 channels abundancy, and axonal length. Finally, the electrophysiological study was designed to study the capability of the motor nerve terminal to improve its output under different nerve stimulation frequencies in control and SMA symptomatic mice.

### 4.1. Mouse Models

For ex vivo studies, pairs of SMA carrier mice (*SMN^+/−^; SMN2^+/+^; SMNΔ7^+/+^*) on an FVB/N background were bred to obtain experimental mice and maintain the colony. Identification of control (*SMN^+/+^; SMN2^+/+^; SMNΔ7^+/+^*) and SMA (*SMN^−/−^; SMN2^+/+^; SMNΔ7^+/+^*) mice was done by PCR genotyping of tail DNA, as previously described [59]. Experimental mouse age was either postnatal day 9–11 (P9–11) or 2–3 months (adults, only controls). All electrophysiological experiments were carried out according to the guidelines of the Directive of the European Council for Laboratory Animal Care and the Animal Care and Ethics Committee of the University of Seville and the Junta de Andalucia. For the cell culture experiments, *SMN^+/−^; SMN2^+/+^* (control) and *SMN^−/−^; SMN2^+/+^* (SMA) mice were used and genotyped as previously described [59]. The mouse line was housed in the central animal facility of the University of Würzburg, and all described procedures and experiments were approved by the animal care and ethics committees and by the Bavarian state authorities.

### 4.2. Primary Embryonic Motor Neuron Cell Culture

For primary embryonic motor neuron cultures, the murine lumbar part of the spinal cord was dissected from control and *SMN^−/−^; SMN2^+/+^* embryos on embryonic day (E) 12.5. After 0.1% trypsin (Worthington) digestion and trituration, the neurons were enriched via anti-p75^NTR^ antibody panning (MLR2, Biosensis M-009-100, Thebarton, Australia) and plated onto poly-DL-ornithine hydrobromide (0.5 mg/mL, P8638, Sigma, Darmstadt, Germany) and laminin-221/211 (2.5 µg/mL, CC085, Merck, Darmstadt, Germany) coated glass coverslips for immunocytochemical investigation and on 35 mm high µ-dishes (81156, Ibidi, Gräfelfing, Germany) for Ca^2+^ imaging. Motor neurons were cultured for five to seven days in Neurobasal medium (Thermo Fisher, Waltham, MA, USA) supplied with 500 µM Glutamax (Thermo Fisher, Waltham, MA, USA), 2% B27 (Thermo Fisher, Waltham, MA, USA), 2% heat-inactivated horse serum (Linaris, Dossenheim, Germany), neurotrophic factors CNTF (5 ng/mL), and BDNF (2 ng/mL) with and without 10 µM nifedipine. The medium was exchanged after 24 h and then every two days.

### 4.3. Immunocytochemistry of Primary Cultured Embryonic Motor Neurons 

For the immunocytochemical investigation of Ca^2+^ channels and actin abundancy at growth cones, five days in vitro (DIV5) motor neurons, or DIV7 for axon length, respectively, were fixed with methanol-free 4% formaldehyde for 10 min and washed thrice with PBS. Permeabilization was performed with 0.3% Triton X-100 for 20 min before cells were washed thrice and incubated with 10% BSA in TBS-T for 1 h at room temperature (RT). The primary antibodies polyclonal, guinea pig anti-Ca^2+^ channel N-type alpha-1B (Ca_v_N) (1:500, Synaptic Systems Göttingen, Germany, 152 305), and monoclonal mouse anti-β-Tubulin I + II (1:1000, Sigma Aldrich St. Louis, MO, USA, T 8660) were incubated in 1 % BSA in TBS-T overnight at 4 °C. On the next day, following three washing steps, the motor neurons were incubated with the following secondary antibodies: Alexa Fluor™ 488 goat anti-mouse IgG, IgM (H + L) (1:500, Thermo Fisher, Waltham, MA, USA, A-10680), Cy™3 AffiniPure donkey anti-guinea pig IgG (H + L) (1:500, Jackson Immunoresearch, 706-165-148) in 1% BSA in TBS-T for 1 h at RT. For F-actin visualization, ActinGreen™ 488 ReadyProbes™ Reagent (1:50, Thermo Fisher, Waltham, MA, USA, Ely, UK, R37110) was incubated with the secondary antibodies. Afterward, the neurons were washed thrice with TBS-T and mounted on glass microscope slides using Aqua-Poly/Mount (Polysciences Warrinton, PA, USA). 

### 4.4. Ca^2+^ Imaging of Primary Cultured Motor Neurons 

For Ca^2+^ imaging, DIV5 motor neurons were incubated with 5 µM Oregon Green™ 488 BAPTA-1 AM (Thermo Fisher, Waltham, MA, USA, in Pluronic™ F-127, Thermo Fisher Scientific) for 15 min at 37 °C. Afterward, motor neurons were washed thrice, incubated with Ca^2+^ imaging buffer (135 mM NaCl, 1 mM MgCl_2_, 10 mM HEPES, 1 mM CaCl_2_, 6 mM KCl, 5.5 mM glucose, pH 7.4), and placed into Tokai Hit stage incubator. Imaging was performed at 37 °C with a constant 5% CO_2_ supply at the Nikon’s Eclipse TE2000 inverted epifluorescence microscope, equipped with a plan APO VC 60x/1.4 NA objective, a perfect focus system, and the ORCA Flash 4.0 V2 C11440-22C camera (Hamamatsu Photonics, Hamamatsu-city, Japan). Using the NIS-Elements AR 4.40.00 software, 16-bit; Nikon, Tokyo, Japan; 1024 × 1024 pixels (2 × 2 binning) pictures were taken at a frequency of 2 Hz over 5 min with an exposure time of 100 ms. Analysis of respective plots was performed with Fiji. The z-axis fluorescence profile of the growth cones was first normalized against the average of the first 20 frames (F/F_0_) and Ca^2+^ transients were counted with the help of the BAR plugin. 

### 4.5. Neuromuscular Preparation

For the electrophysiological experiments, the *transversus abdominis* (TVA) muscle was dissected with its nerve branches intact and fixed with 0.2 mm pins in a Petri dish with the base covered with a silicone elastomer (Sylgard), as previously described [20]. The dissection was carried out under a stereoscopic microscope. The preparation was kept in a physiological solution: 135 mM NaCl, 5 mM KCl, 2 mM CaCl_2_, 2 mM MgCl_2_, 12 mM NaHCO_3_, and 20 mM glucose. This solution was continuously gassed with carbogen (95% O_2_ and 5% CO_2_) to maintain the pH around physiological pH (approximately 7.35).

### 4.6. Electrophysiological Experiments and Drugs 

For the ex vivo functional study, the dissected muscle was pinned in a 3 mL methacrylate recording chamber with a Sylgard base and a solution exchange system. Recordings were done at RT. Components of the experimental setup included a custom-made nerve suction electrode, isolated pulse stimulator (Model 2100, AM-Systems, Carlsborg, WA, USA), intracellular recording amplifier (TEC-05X, Npi electronic GmbH, Tamm, Germany), upright microscope (BX50WI, Olympus Spain), micromanipulators (Narishige, MHW-3, Japan), interface (PowerLab 4SP, ADInstruments, Sydney, Australia), computer, signal acquisition and monitoring software (Chart5, ADInstruments, Australia), anti-vibration table (Newport, CA, USA), and Faraday cage. Before recordings, the neuromuscular preparation was incubated for 20–30 min with µ-conotoxin GIIIB (2–4 µM; Alomone Labs, Jerusalem, Israel), a specific blocker of muscle voltage-gated sodium channels, to inhibit muscle contraction. In the presence of the blocker, single muscle fibers near motor nerve endings were impaled with glass microelectrodes (10–20 MΩ) filled with 3 M KCl and connected to the intracellular recording amplifier. The nerve stimulation consisted of square wave pulses of 0.15 ms duration. The minimum stimulation threshold amplitude was estimated, and this value was taken twice as a reference for subsequent stimulation. Unless otherwise stated, stimuli were applied at 0.5 Hz for 200 s and 20 Hz for 5 s (by triplicated trains) and evoked EPPs and mEPPs were recorded. Before analysis, the amplitudes of the EPP and mEPPs recorded at each NMJ were linearly normalized to −70 mV resting membrane potential and EPPs were corrected for nonlinear summation [60].

The drug used to test the modulation of neurotransmitter release were nifedipine (10 and 50 μM; Alomone Labs), W-7 (10 μM; Santa Cruz Biotecnology, Dallas, TX, USA), verapamil (3 μM; Merk, Spain), nimodipine (10 and 50 μM; Tocris Bioscience, Bristol, UK), and nitrendipine (1, 25 and 50 μM; Merk, Spain). Nifedipine, nimodipine, nitrendipine, and W-7 were dissolved in DMSO with a maximal final concentration DMSO of 0.1%, which did not affect secretion [20]. Verapamil was dissolved in water. The experimental procedure was as follows: First, six fibers per muscle were recorded without drugs. Then, the preparation was incubated for 20–30 min with the drug of interest, and then six fibers were recorded. In experiments where a second drug was subsequently applied, the solution was not changed, but the second drug was added and incubated for 20–30 min. Subsequently, six fibers were recorded in the presence of both substances. 

### 4.7. Statistical Analysis

Results are presented as mean ± SEM. Statistically significant differences were determined using the parametric Student’s *t*-test and the non-parametric Mann–Whitney U test for those experiments in which fibers were recorded in the absence and presence of a drug; the parametric ANOVA test of one factor was used with the Bonferroni test for multiple comparisons and the non-parametric Kruskal–Wallis test was used in those experiments in which fibers were recorded both in the absence and the presence of a drug, as well as during the combination of two drugs.

## 5. Conclusions

Ca^2+^ signaling is essential for normal neuronal development and neurotransmitter release. In SMA mouse models, motor neuron development and neurotransmission are impaired and cell Ca^2+^ regulation is defective. Positive modulation of Cav2 channel activity by modifier agents shows beneficial effects in these models. In recent years, an enormous advance in SMA treatment of patients has been achieved by SMN-restoring therapies; however, early intervention is critical. Thus, developing gene-independent additional therapies is essential to reach the best results. Here, we studied the effect of nifedipine on SMA. Nifedipine, a classical antagonist of Cav1.2-type channels, also acting on other targets intervening in Ca^2+^ regulation, has been shown to increase neurotransmitter release at the NMJ of disease-unaffected animals and protect motor neurons from different insults. Our results indicate that nifedipine benefits the development of SMN-deficient motor neurons in vitro and partially enhances neurotransmission at already altered NMJ in SMA mice. We propose that nifedipine may help to regulate motor function in SMA in patients treated with SMN enhancers. Future preclinical studies may explore this possibility.

## Data Availability

Data are available upon request.
